# Recreational Skydiving—Really That Dangerous? A Systematic Review

**DOI:** 10.3390/ijerph20021254

**Published:** 2023-01-10

**Authors:** Christiane Barthel, Sacha Halvachizadeh, Jamison G. Gamble, Hans-Christoph Pape, Thomas Rauer

**Affiliations:** 1 Department of Trauma Surgery, University Hospital Zurich, 8091 Zurich, Switzerland; 2 St. George’s University School of Medicine, St. George, Grenada

**Keywords:** skydiving, parachute, injuries, fatality, systematic review

## Abstract

Skydiving have gained mainstream popularity over the past decades. However, limited data exist on the injury risk or type associated with skydiving. This systematic review evaluated the injuries and fatalities of civilian skydivers. A PRISMA-guided literature search was performed in MEDLINE, Web of Science, Cochrane Library, and Embase using the following MeSH terms: “skydiving” or “parachute” alone or in combination with “injury” or “trauma” was performed including all studies through June 2022 in both English and German. Additionally, injury reports from the German, American, and British Parachute Associations were reviewed. Of the 277 articles matching the selected search terms, 10 original articles and 34 non-scientific reports from various skydiving associations were included. More than 62 million jumps were evaluated, with an average of 3,200,000 jumps per year, which showed an average injury rate of 0.044% and an average fatality rate of 0.0011%. The most common injuries sustained by recreational skydivers involved the lumbar spine and lower extremities. Injuries were most commonly reported during the landing sequence. With modern equipment and training methods, fatalities occur in less than 1 per 100,000 cases, and serious injuries requiring hospitalization in less than 2 per 10,000 cases. This puts the assessment of skydiving as a high-risk sport into perspective.

## 1. Introduction

Accidental injury causes more than 9% of deaths worldwide, approximately five million people, each year [1]. In developed countries, sport-related incidents account for a large proportion of lethal and non-lethal injuries. In Switzerland, more than 250,000 occupational injuries and 550,000 non-occupational injuries are reported annually [2]. A total of 36% of non-occupational accidents occur in sports settings, almost 200,000 injuries each year. Of these sports-related injuries, about 65% occur during ball sports and winter sports, the mechanisms of which are well-researched. This makes the potential risks more transparent and informed decision-making and risk mitigation possible. However, only limited information exists regarding accidents and injuries related to parasports (activities with a controlled descent using some kind of deceleration device) [2]. Among all parasports, the most reported injuries are associated with recreational skydiving [3]. Skydiving is often described as a high-risk or even lethal activity but limited data exist regarding the true risk of injuries connected with recreational skydiving.

In the literature, the terms “extreme sports” and “high-risk sports” are often used interchangeably [4]. A clear definition of the term “high-risk sport” seems to be very problematic due to the complexity of the included aspects. Therefore, in the present study, we refer to the definition of “high-risk sport” formulated by Breivik et al. in 1994 as a sport in which athletes must accept the possibility of serious injury or death as an immediate direct component of the sporting activity [4].

During a skydive jump, injuries can occur at three different phases: the exit phase, opening phase, and landing phase. Exit from the airplane can result in minor lacerations and contusions. After deploying the parachute, also known as the opening phase, the skydiver experiences average decelerations of 3–5 times the Earth’s gravitational acceleration [5]. Landing is challenged by a complex interaction of speed, flying technique, type of parachute, weather, and geographical conditions. Thus, objectively assessing the generalizable risks associated with recreational skydiving requires comprehensive data from a wide population of skydivers.

The objective of this systematic review was to answer the following research questions:

(1) What are the most common injuries discussed in current literature?
(2) What is the frequency of injuries in relation to the number of completed jumps?
(3) In what phase of the jump do most of the injuries occur?

## 2. Materials and Methods

Following the PRISMA Guidelines for systematic reviews, we searched the databases MEDLINE, Web of Science, Cochrane Library, and Embase with the following MeSH terms: “skydiving” or “parachute” alone or in combination with “injury” or “trauma”. Additionally, we reviewed injury reports from the following skydiving associations: the German Parachute Association (Deutscher Fallschirmsportverband), United States Parachute Association, and British Parachute Association. We searched all documents from beginning to present with a strong emphasis on reports from the last 20 years.

Inclusion criteria: Original articles, English or German language, injury-reports, and reviews. All data available through June 2022 were included in this analysis.

Exclusion criteria: Non-injury-related articles (e.g., endocrinological or psychiatric studies), extreme parachute sports (e.g., BASE jumping), static line parachute, or military reports. The well-characterized injury and accident risks in military skydiving are likely not generalizable to recreational skydiving, as military skydiving is mainly performed in lower altitude, under worse weather conditions, using round parachutes, and a static line system. Because of these critical differences between military and recreational skydiving, these types of studies were excluded from this review. Furthermore, case reports, studies evaluating reporting systems, studies on audiological system assessment, and articles examining the costs to national health systems were excluded. Additionally excluded were one article discussing risks for pregnant parachutists and one article discussing a potential problem for women deploying the parachute as a result of comparatively less upper body strength—a non-issue with modern deployment mechanisms (modern bridles are easy to pull and only minimal physical strength is required).

First line studies were ranked according to topic, study size and type, country, and year of publication. Studies were then allocated to four subcategories: fatalities, prevalence, type of injury, and phase of jump. Factors also noted from each study include: (1) Prevalence and fatality—are data only for lethal accidents? (2) Are minor injuries such as contusions and abrasions also included or only injuries requiring medical treatment? (3) Are statistics reported in relation to the number of certified divers or performed jumps? (4) Are all phases of the jump considered or does the study only assess one phase (usually the landing phase)?

Furthermore, we included statistics on fatalities and reported injuries, which were extracted from the reports or minutes of the Deutscher Fallschirmsportverband, United States Parachute Association, and British Parachute Association. The British Parachute Association holds bimonthly meetings in which incidents are reported. Incidents are categorized into injuries, malfunction/deployment issues, ‘off landings’, and reports involving aircrafts. Incidents are further categorized by the diver’s level of training: student, “A” License parachutists or above, and tandem jumps. A combined report is available containing data since 1998.

The Deutscher Fallschirmsportverband e.V. (DFV) publishes an annual report on fatalities and serious injuries with a short explanation about the mechanism leading to the deadly accidents.

The United States Parachute Association publishes an annual fatality report. The number of fatalities is reported in relation to the estimated annual jumps.

The Fédération Aéronautique Internationale (FAI) is an international organization with the basic aim of furthering aeronautical and astronautical activities worldwide. Between 31 and 42 countries report their annual fatalities to this organization (an annual average of 6.2 mil jumps). We did not use these data specifically because the data of the BPA, USPA, and DFV were included and the other sources were not conclusively clear and constant.

**Figure 1:**
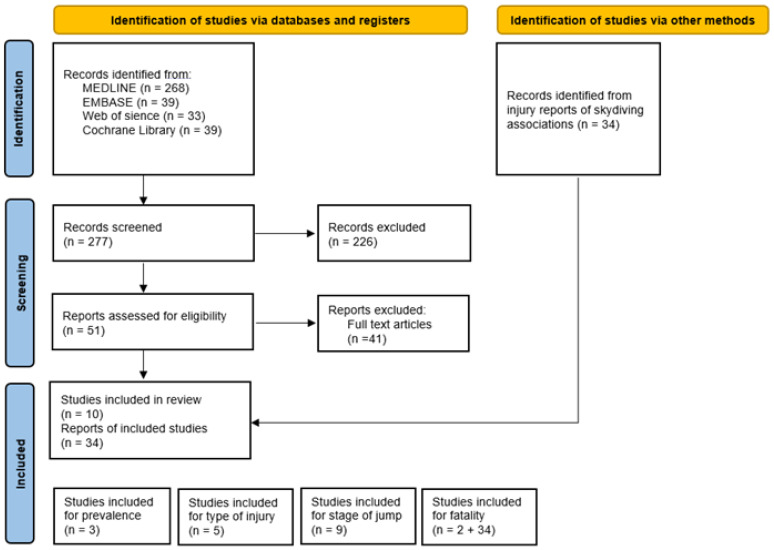
PRISMA flow diagram for study selection.

Of the 277 original articles, 51 (18.4%) articles met the inclusion criteria. Ten (3.6%) articles were excluded as case reports describing single parachuting incidents and 30 were excluded due to the following exclusion criteria: 15 were military reports, 13 did not include skydiving injuries, one was about the extreme sport base jumping, and one was on static line parachuting. Additionally, one article was unavailable. The remaining 10 studies, which are listed in Table 1, Table 2 and Table 3, were allocated to one or more of the four main categories—fatalities, prevalence, type of injury, and phase of jump. The 34 reports from various skydiving associations were additionally included in the “fatalities” category. The selection process is summarized in Figure 1.

## 3. Results

In total, nearly ten million jumps were reviewed with a mean of 610,000 jumps per year with a risk of injury of 0.044% and a risk of fatality of 0.0011%.

### 3.1. Most Common Injuries

A total of five studies were included, which described the most common injury types sustained (Table 1). The Swedish Parachute Association maintains SKYNET, a national register of skydiving injuries and one of only a few trauma databases available for research on sport parachuting. A descriptive epidemiological study of reported injury events (n = 257) in Swedish skydiving over a 5-year period (1999–2003) revealed an incidence of 48 per 100,000 jumps. Most injuries occurred to the lower extremities (51%), upper extremities (19%), and spine (18%) [6]. This distribution of injuries to the lower and upper extremities and spine was similar to a Danish study of 110,000 sports jumps published 20 years earlier by Ellitsgaard et al. [7].

In New Zealand, the Auckland City Hospital Trauma Registry was used to identify all patients admitted with serious injury as a result of parasport incidents over an 8-year period. The analysis of demographic and injury-related data revealed that skydiving was responsible for 66% of the 38 serious parasport injuries. Fractures of the lower limbs (47%) and lumbar spine (19%) were the predominant injuries, mainly attributed to misjudgment of the landing speed and altitude [3].

During two World Freefall Skydiving Conventions, data from 8976 skydivers (117,000 jumps) revealed that most of the 204 skydiving related injuries were minor injuries such as abrasions/contusions or lacerations (53.5%), 28.4% were injuries to the extremities, and 6.9% to the spine [8].

### 3.2. Jump Phase

We identified a total of nine studies that detailed injuries sustained during the jump phase (Table 2). One detailed analysis of accident occurrence separated by jump phase was carried out by Westman in 2007 [6]. He separated the jump in to four phases and the analysis revealed that by far the greatest incidence of injury occurred during the landing phase of the parachute flight: 1—aircraft exit (2%), 2—freefall (2.7%), 3—parachute opening (7.4%), 4—parachute flight/landing (87.9%).

A survey of musculoskeletal pain experiences in 658 sport skydivers revealed that neck pain, seemingly related to the opening phase, is a common experience [9].

Unlike military skydivers, most injuries in civilian skydivers occur during the first jump—probably because civilian jumpers receive less training than military skydivers (6.5 h compared to 31.5 h) prior to their first independent jump [6]. The lower injury rates for civilian skydivers in subsequent jumps are likely influenced by the optimization of time and location for ideal weather and visibility conditions in recreational jumps. This is not always the case for military skydivers who may land in less-than-ideal conditions more frequently than their civilian counterparts. An alternative explanation, however, could be that military skydivers do not have the option of quitting jumping after their first jump if they suffer a minor injury, so perhaps the proportion of first jumps is higher in civilian populations.

Another study analyzing injuries over a 5-year period in charity parachuting events revealed as many as 95% of the skydivers who experienced injuries were first-time jumpers [10]. However, the skydivers investigated in this study jumped with static parachutes from low altitude. Therefore, the results of this study cannot be directly applied to modern skydiving, since modern recreational skydiving does not use round parachutes and rarely uses jumps with static lines from low altitude.

The United States Parachute Association (USPA) collects and publishes data concerning the reported fatalities in skydiving. In 2002, Griffith and Hart categorized the causes of skydiving fatalities from 1993 to 1999 [11]. Incorrect procedures (31%) and landings (27%) were the most common causes. The same summary for the period of 2000 to 2004 showed landings (35%) as the most common cause, followed by midair collisions (18%) and incorrect procedures (15%) [12]. Further analysis of the USPA data on fatalities from 1992 to 2005 revealed that low turns to the ground (dives in order to gain a higher speed—so called hook turns) are a potential major cause of death [13]. In this analysis, fatalities caused by parachute malfunction had decreased, but an associated “rise in landing-related skydiving fatalities” was noted [14]. This study postulated that the “swooping” landing style might be an underlying reason for this observation.

**Table 2:** Studies included for injury analysis by jump phase.

Title	Author	Country	Year	PMID	Stage	Subjects	Jumps
Injuries in Swedish skydiving	Westman A [6]	Sweden	2007	17224436	Landing	n/a	539,885
Serious parasport injuries in Auckland, New Zealand.	Christey GR [3]	Australia	2005	15796732	Landing	38	n/a
The epidemiology of skydiving injuries: World Freefall Convention, 2000–2001	Barrows TH [8]	USA	2005	15657007	Landing	8976	117,000
Parachuting injuries: A study of 110,000 sports jumps	Ellitsgaard N [7]	Denmark	1987	3580720	Landing	143	110,000
Are hook turns a major obstacle to safe skydiving? A study of skydiving fatalities in the United States from 1992 to 2005	Vidovic M [13]	USA	2007	18229535	Landing	439	n/a
An analysis of U.S. parachuting fatalities: 2000–2004	Hart CL [12]	USA	2006	17326520	Landing	125	n/a
A summary of U.S. skydiving fatalities: 1993–1999	Griffith JD [11]	USA	2002	12186229	Incorrect procedures	241	n/a
Fatalities in Swedish skydiving.	Westman A [6]	Sweden	2005	16039597	No/Low reserve activation	n/a	2,176,471
Rise in landing-related skydiving fatalities	Hart CL [12]	USA	2003	14620223	Landing	n/a	n/a

**Table 3:** Studies included detailing the prevalence of accidents.

Title	Author	Country	Year	PMID	Subjects	Jumps	Prevalence of Any Injury during a Jump
Injuries in Swedish skydiving	Westman A [6]	Sweden	2007	17224436	n/a	539,885	0.0476%
The epidemiology of skydiving injuries: World Freefall Convention, 2000–2001	Barrows TH [8]	USA	2005	15657007	8976	117,000	0.1740%
Parachuting injuries: A study of 110,000 sports jumps	Ellitsgaard N [7]	Denmark	1987	3580720	143	110,000	0.1409%

### 3.3. Prevalence of Accidents and Fatalities

Data from 8976 skydivers, totaling 117,000 jumps, were collected during two World Freefall Skydiving Conventions [8]. In this period, 204 (2.3%) athletes were treated for injuries; a total injury rate of 0.174% (injuries/jump). Most of these injuries were minor (66%). Injuries requiring treatment in the emergency department occurred at a rate of 0.06%. The rate of hospitalization was 0.018% and one fatality was reported. Table 3 lists the three studies included regarding the accident prevalence across three countries.

Two large studies investigated the fatality rate of recreational skydiving. In 1987, a Danish study observed 110,000 jumps performed in the period from 1979 to 1983 and reported an injury prevalence of 0.14% and a fatality rate of 0.005% [7]. A Swedish study investigated approximately two million jumps over a 40-year period (1964–2003) with an overall fatality rate of 0.0017% throughout, but only 0.0008% for the most recent decade (1994–2003) [15].

**Figure 2:**
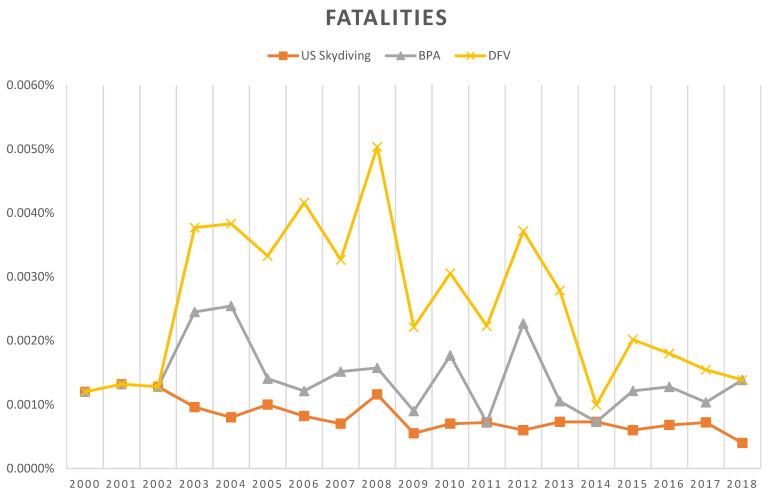
Comparison of the fatality rates by country over a 19-year reporting period (2000–2018).

Fatality rates over the last 15 years from the British Parachute Association resemble the U.S. figures, which are lower than the German numbers (0.0006% and 0.0008% vs. 0.0015%; Table 4). In the last decade, the reported number of fatalities in Germany has decreased and the numbers have equalized (fatality rates in 2017: BPA 0.0003%, USA 0.0007%, and DFV 0.0005%; Figure 2).

## 4. Discussion

Skydiving is generally considered as a high-risk sport. However, no systematic reviews exist that have summarized the injuries and fatalities due to skydiving in civilian populations. Our study revealed the following results:

1. The most common injuries were to the lumbar spine and lower extremities;
2. Injuries occur at a rate of approximately 0.03% to 0.17% and fatalities in 3–10 fatalities per 1 million jumps;
3. Injuries occur most commonly in the landing sequence.

The results of our systematic review were confirmed in a recent prospective study of the epidemiology of deaths and injuries associated with skydiving in France, in which 83.3% of injuries occurred during the landing sequence and 64.3% of injuries involved the lower extremities [16]. Most of the available data have focused only on lethal injuries. There are a few published medical reviews of non-lethal injuries. From the five studies regarding injury distribution, only one [8] observed all jumps during a time period (World Freefall Convention) and also recorded minor injuries. Minor injuries such as bruises or high ankle sprain are often treated without medical intervention and are often not reported to an insurance company or a skydiving association. Most studies analyzing skydiving injuries such as that by Westman and Björnstig in 2007 only covered reported injuries [6]. As was the case with the large Danish study researching 100,000 jumps—only fatalities and injuries receiving medical treatment were registered [7]. Most minor injuries, that is, injuries with an Abbreviated Injury Score of 1 such as superficial lacerations or ankle strains, are not reported and would be missed by these studies, as was the case in many other sports reviewed. Even the validated reporting system, SKYNET, was found to have a low sensitivity for minor injuries [17]. In 2011, a web-based questionnaire was developed and piloted with 102 skydivers [18]. A wide-ranging study using data from this questionnaire was not found.

Missing data for minor injuries from some studies may explain the wide range in injury prevalence between studies. Barrows’s study, which included injuries that did not require medical treatment, revealed an incident of 0.174% [8], while other studies showed much lower incidences (e.g., 0.047% in the 2007 study by Westman and Bjornstig) [6]. Reported incidents at the British Parachute Association averaged 0.0652% and for the Deutscher Fallschirmsport Verein, it was 0.0251%. A stronger understanding of minor injuries, their causes, and mechanisms may help identify and emphasize ways that skydivers can mitigate or even prevent such injuries with the use of commonplace protective equipment such as helmets, kneepads, and ankle stabilizers. Military skydivers are equipped with specialized ankle braces shown to reduce the risk of ankle injuries by 40–50% [19].

Regarding non-minor injuries, extremity, especially lower extremity, and spine injuries are the most common. At first glance, this seems intuitive due to the effects of high velocity ground impact on the body. However, taking a closer look at the causes of hard ground contact revealed that different mechanisms (e.g., parachute entanglements, collisions, or other equipment malfunction) lead to an increased fall velocity. While unbraked falls are usually fatal, landing accidents are more diverse. The normal landing sequence consists of a special landing pattern (downwind, crosswind, and final approach) that aims to land into the wind. Shortly before ground contact, the forward motion of the parachute is slowed down by a strong pulling of the two toggles, which slows down the descent for landing. This maneuver is called “flare”. Cases with insufficient braking result in a predictable trauma pattern: the drag of the parachute pulls the body to an upright position and the major impact occurs through the lower extremities, which is then transmitted to the pelvis and spine. If the incident occurs after normal braking during the landing, a more variable trauma mechanism occurs. This can be anything from a simple ankle sprain to flips on the ground (with possible neck or facial trauma) to collision with an object.

In published medical reports, injury frequency statistics have primarily been available only for lethal injuries. In past decades, parachute deployment was identified by these statistics as a leading cause of lethal injuries. Improvements in the deployment mechanisms in modern parachutes correlate with a significant reduction in the number of lethal injuries resulting from parachute deployment failure (e.g., during 1994–2003 the fatality rate of skydivers in Sweden was 11 times lower than 1964–1973) [15]. An analysis of data from 507 skydiving fatalities in the USA between 1986 and 2001 indicated that there was an increase in parachute-landing deaths associated with the new high-performance parachutes and aggressive flying techniques [14]. The total number of fatalities remained fairly stable at 0.0074% between 1993 and 1999 and decreased in the past two decades to a level between 0.0004% and 0.001% (Parachute Associations) [12,14]. The German Parachute Association reports the greatest reduction in fatal incidences in the past decade. This might be related to improved training developed in the U.S. in the early 1980s. This “Accelerated Freefall” (AFF) training became common practice first in the U.S. and GB, and later in the German speaking countries. Before this more recent method of training, skydiving students had to start with a round parachute on a static line system. However, in the AFF training, a steerable “square” parachute is used and the training starts directly with a solo flight where two instructors jump with the student during their first three jumps. Once the student has demonstrated the required skills, the student is advanced to the next level. In this way, closer monitoring is assured.

We found only one large study quantifying the risk of skydiving injuries during the World Freefall Convention 2000–2001, with a total injury rate of 17.4 injuries per 10,000 jumps [8]. Improved equipment, regulations, and safety procedures since the time of this study have likely altered the safety issues identified by this study 20 years ago, one such example being the aforementioned AFF basic training.

The prevalence of injuries has also been shown to be related to the level of experience a skydiver has. In the Swedish study from 1964–2003, inexperienced skydivers had the highest risk of a fatal outcome [15]. Unstable body position in freefall (causing a line entanglement after parachute activation or a parachute activation failure) and unintentional water landings were the main causes of accidents with the old training methods.

Most data available regarding injury mechanisms or phase of the jump come from case reports. Eleven relevant case reports were found, four of which described injuries to the spine, three described vascular events, and two described scapula/clavicle fractures. Of the spine injuries, two reported sudden hyperextension of the neck during the parachute opening sequence: one traumatic hangman’s fracture [20] and one cervical disc herniation in an occupational skydiver [5]. The other two spine injuries occurred during landing—one because of an incorrect tandem landing [21] and one during a hard landing after a collision with another jumper close to the ground [22]. Two case reports described traumatic vessel dissections—an internal carotid artery dissection with delayed stroke after a traumatic landing [23] and a dissection of the thoracic aorta (among multiple life threatening injuries) after a failed parachute deployment [24]. One skydiver experienced multiple facial hemorrhages and dizziness due to the deceleration during the parachute opening sequence [25]. Injuries to the shoulder belt were described for two skydivers—one experienced bilateral scapula fractures while exiting the plane [26] and another a rare fracture of the medial clavicle [27].

In general, injuries associated with skydiving can occur at different phases of a jump: aircraft exit, free fall, parachute opening, parachute flight, or landing. In a typical skydive, the athlete exits the aircraft at around 4000 meters above ground level (mAGL) and falls for 45–90 seconds with a terminal vertical velocity of approximately 200 km/h [27,28]. At approximately 1000 mAGL, the parachute is deployed and slows the skydiver to around 20 km/h within a few seconds. This abrupt deceleration is called parachute opening shock [28]. Rocks et al. investigated the forces acting on the parachutist at the time of the parachute opening shock. They were able to show that at the time of the parachute opening shock, the mean (±SD) resulting accelerations and angular velocities were 5.8 (±1.6) g and 255.9 (±74.2) degrees per second (deg/s), respectively, for the head and 4.3 (±1.5) g and 181.3 (±61.2) deg/s, respectively, for the body [29]. We found two articles about injuries happening in this special moment of the jump—a case report about a hangman’s fracture [20] and an analysis of musculoskeletal pain [30]. Here surface electromyography (EMG) during jumps revealed anticipatory muscle tensing, especially of the neck muscles, at the time of parachute opening, which increases the risk of musculoskeletal injury [30].

One study had injury data clearly distributed to the jump stages [6]. Furthermore, Westman summarized turbulence, strong wind, miscalculation during an ordinary flight, low landing turns, parachute traffic disturbance, entanglement, and the faster sink rate of the reserve parachute as potential factors of the parachute flight that contribute to incidents resulting in injury [6]. Trauma mechanisms and injury patterns differ according to the jump phase and should be subdivided into aircraft exit, parachute opening sequence, parachute flight, or landing-related for future studies. Modern stunts such as hook turns are currently a major cause of injuries in the setting of the increased overall safety of this previously “high-risk” parasport. This trend should be closely monitored in the coming years.

### Limitations

Apart from military studies, only Swedish and American reports have provided the few comprehensive studies in existence. Datasets, especially about fatalities in skydiving, are provided by many individual organizations. We only used the data of the biggest three: BPA, USPA, and DF, as these data were documented in a comprehensible manner.

The collected figures of the FAI could be considered if further data about the source are provided. We did not use the data of the FAI specifically because the data of BPA, USPA, and DFV were included, and the other sources were not conclusively clear and constant.

However, skydiving has gained international popularity and is practiced all over the world. Due to local variations in weather and geographical conditions, these data may have limited generalizability. Furthermore, reports from thirty or forty years ago have minimal applicability to the current risk of this parasport due to an ongoing evolution in training and equipment.

Inconsistencies exist between studies due to the lack of a standardized reporting system, with minor injuries often going unreported or underreported. These differences make comparisons between studies difficult and statistical meta-analysis impossible.

## 5. Conclusions

With modern equipment and training methods, fatalities occur in less than 1 per 100,000 cases and serious injuries requiring hospitalization in less than 2 per 10,000 cases. This puts the assessment of skydiving as a high-risk sport into perspective, especially considering the definition of high-risk sport as a sporting activity in which athletes must accept the possibility of serious injury or death as an immediate part of the sporting activity, especially when comparing these results with other so-called high-risk sports. For example, amateur boxers suffer an injury on average every 2.5 h in competition and every 772 h in training [31], or one in three recreational surfers suffers a traumatic injury within 12 months [32].

Regarding skydiving, the data have been carefully collected and curated regarding fatal accidents for several decades, which has led to improvements in the equipment and training at key points identified as potential sources of risk. These data are lacking for non-fatal accidents. To objectively assess the injury risks associated with skydiving, there is a need for further studies investigating the epidemiological, geographical, and clinical aspects of injuries among skydivers combined with the weather conditions and the level of experience of the individual athlete. A detailed investigation of the mechanism and timing of injuries would help skydiving instructors as well as manufacturers of skydiving equipment to improve on the techniques and materials. An extension of the existing Swedish questionnaire could be used to collect these data on an international level. Producing a standardized reporting system including the incidence and injury severity numbers should be attempted in order to identify areas for improvement in parasport safety.

## Figures and Tables

**Table 1:** Studies included for most common injury distribution.

Title	Author	Country	Year	PMID	1. Most Injury	2. Most Injury	3. Most Injury	Subjects	Jumps
Injuries in Swedish skydiving	Westman A [6]	Sweden	2007	17224436	51% lower extremities	19% upper extremity	18% spine	n/a	539,885
Parachuting injuries: a study of 110,000 sports jumps	Ellitsgaard N [7]	Denmark	1987	3580720	59.7% lower extremities	17% upper extremity	10% spine	143	110,000
The epidemiology of skydiving injuries: World freefall convention, 2000–2001	Barrows TH [8]	USA	2005	15657007	47% extremity injury	17.1% back injury	12.9% lacerations	8976	117,000
Serious parasport injuries in Auckland, New Zealand.	Christey GR [3]	New Zealand	2005	15796732	47% lower limb	8% upper extremity	6% thoracic spine	38	n/a
Musculoskeletal pain and related risks in skydivers: a population-based survey	Nilsson J [9]	Sweden	2013	24261055	25% neck region	16% shoulder	10% thoracic spine	658	n/a

**Table 4:** Injuries reported by the British (BPA) and German (DFV) Parachute Associations.

Reported Injuries						
	BPA			DFV		
Year	Jumps	Reported Injuries		Jumps	Reported Injuries	
2003	268,816	242	0.0900%	302,760	77	0.0254%
2004	229,565	180	0.0784%	310,650	98	0.0315%
2005	245,472	175	0.0713%	260,800	65	0.0249%
2006	253,994	219	0.0862%	271,500	91	0.0335%
2007	244,838	200	0.0817%	286,000	87	0.0304%
2008	240,426	165	0.0686%	289,200	77	0.0266%
2009	286,794	206	0.0718%	304,700	85	0.0279%
2010	280,752	223	0.0794%	311,245	74	0.0238%
2011	286,840	199	0.0694%	331,410	93	0.0281%
2012	299,921	168	0.0560%	345,430	95	0.0275%
2013	309,529	163	0.0527%	346,815	82	0.0236%
2014	344,607	168	0.0488%	374,545	90	0.0240%
2015	325,254	154	0.0473%	374,620	10	0.0027%
2016	333,955	163	0.0488%	385,625	96	0.0249%
2017	315,290	142	0.0450%	393,200	83	0.0211%
2018	303,882	144	0.0474%	-	-	-

## Data Availability

The raw data used in the analyses of this study are available in the authors’ database.
